# Pathogenic Role of IL-17-Producing Immune Cells in Obesity, and Related Inflammatory Diseases

**DOI:** 10.3390/jcm6070068

**Published:** 2017-07-14

**Authors:** Marwa Chehimi, Hubert Vidal, Assia Eljaafari

**Affiliations:** 1 Univ-Lyon, CarMeN Laboratory, Inserm U1060, INRA U1397, Université Claude Bernard Lyon 1, INSA Lyon, Charles Mérieux Medical School, F-69600 Oullins, France; marwa.chehimi@gmail.com (M.C.); hubert.vidal@univ-lyon1.fr (H.V.); 2 Clinical Research Department, Hospices Civils de Lyon, Centre Hospitalier Lyon-Sud, F-69495 Pierre Bénite, France

**Keywords:** obesity, IL-17-producing T (IL-17) cells, adipose-derived-stem-cells, inflammatory diseases, immuno-metabolism

## Abstract

Obesity is associated with low-grade chronic inflammation. Indeed, adipose tissues (AT) in obese individuals are the former site of progressive infiltration by pro-inflammatory immune cells, which together with increased inflammatory adipokine secretion induce adipocyte insulin resistance. IL-17-producing T (Th17) cells are part of obese AT infiltrating cells, and are likely to be promoted by adipose tissue-derived mesenchymal stem cells, as previously reported by our team. Whereas Th17 cell are physiologically implicated in the neutralization of fungal and bacterial pathogens through activation of neutrophils, they may also play a pivotal role in the onset and/or progression of chronic inflammatory diseases, or cancer, in which obesity is recognized as a risk factor. In this review, we will highlight the pathogenic role of IL-17A producing cells in the mechanisms leading to inflammation in obesity and to progression of obesity-related inflammatory diseases.

## 1. Introduction

Overweight and obesity are defined as excessive fat accumulation induced by consuming many more calories than the body can burn. They have become a major worldwide health problem. In fact, a recent study has evaluated that more than 2 billion adults worldwide are overweight (Body Mass Index (BMI) ≥ 25 kg/m^2^), and 671 million of them are clinically obese (BMI ≥ 30 kg/m²) [1]. Accordingly, a model of mathematical analysis forecasts that more than 51% of the population worldwide will be obese by 2030 [2].

Excessive weight gain can provoke severe complications such as hypertension, type II diabetes (T2D), atherosclerosis and hyperlipidemia, which define the “metabolic syndrome”. But, only half of obese patients with BMI from 30 to 50 kg/m^2^ are metabolically unhealthy [3]. However, transient metabolic syndrome can be encountered in lean individuals, such as during infection, where increased secretion of TNF-α, IL-6 and IL-1β by macrophages inside infected tissues induces a locally and temporary insulin-resistant (IR) state. Such a response is beneficial for the elaboration of a potent defense against pathogens, through a decrease of nutrient storage but an increase of glucose concentrations [4]. While transient inflammation-mediated IR can be helpful, excessive adipocyte death and lipid availability leads to permanent inflammation, and transforms a temporary IR state into a chronic and systemic IR state. Thus, chronic adipose tissues (AT) inflammation is considered the causal event leading to insulin resistance, and subsequently to T2D in obese individuals. At an advanced stage, obese AT become heavily infiltrated by a variety of immune cells such as macrophages and T cells, leading to inflammation. T cell distribution varies in obese AT, with a trend towards higher CD8^+^ to CD4^+^ ratios, in particular in visceral AT [5]. Whereas macrophage activation is likely to be mediated by IFNγ-secreting T helper 1 (Th1) cells, CD8^+^ T cells appear to play an essential role in the initiation of adipose tissue inflammation, through recruitment and differentiation of macrophages [6]. In addition to Th1 cells, Th17 cells, recognized as active players of chronic inflammatory and autoimmune diseases, have been recently found to play an important role in obesity and T2D (as reviewed below).

Because obesity appears to exacerbate chronic inflammatory diseases, autoimmune diseases and cancer [7,8], in this review, we will point out the role of pathogenic Th17 cells in obesity and related chronic inflammatory diseases and cancer. We will also describe the impact of immuno-metabolism interference in Th17 cell differentiation, which should bring new insights into the understanding of obesity contribution in inflammatory disease exacerbation.

## 2. IL-17 Producing Cells

IL-17A or F is produced by cells of innate and/or adaptive immunity. Th17 cells are, however, the major source of IL-17 production, an inflammatory cytokine that in part induces neutrophil recruitment towards inflammation sites, through release of chemokines, like CXC-chemokine ligand-1 (CXCL1), CXCL2, IL-8, or GCP-2/CXCL6 [9]. Moreover, IL-17A or F increases neutrophil elastase and myeloperoxydase (MPO) activities and plays an important role in the defense against the bacteria or fungi that have not been cleared by Th1 or Th2 cells, such as *Propionibacterium acnes*, *Klebsiella pneumoniae*, *Citrobacter rodentium*, or *Candida albicans* [10]. Accordingly, in the hyper-IgE syndrome Th17 cell deficiency, which occurs subsequently to STAT3a mutation, leads to recurrent *C. albicans* and *Staphylococcus aureus* skin and lung infections [11]. Besides physiology, Th17 cells have been given particular attention in pathology, due to their implication in chronic inflammatory/autoimmune diseases, or cancer.

### 2.1. Characterization of Th17 Cells

IL-17A and IL-17F are members of a six cytokine family, i.e., IL-17A to IL-17F, which surface receptors are IL-17RA to IL-17RE. IL-17A (also named IL-17) is the major cytokine secreted by Th17 cells. It forms a homo or heterodimer with IL-17F, and signals through binding to IL-17RA/IL-17RC heterodimeric complex. IL-17RA ubiquitous expression may account for the potential propagation of IL-17-mediated inflammation [12]. Commitment of IL-17 from naive T cells has been shown to require a combination of antigen-presenting cells (APC)-secreted cytokines, such as at least IL-6 and TGF-β, together with CD28 plus ICOS costimulation [13]. While naive T cells do not express the IL-23 receptor, IL-23 is required for differentiation, expansion and maintenance of Th17 cell pools [14]. Differentiation of Th17 cells are derived from a three-step process. In the first step, the combination of TGF-β and IL-6 drives naive T cells towards the Th17 cell pathway. IL-6 through Signal Transducer and Activator of Transcription 3 (STAT3), triggers activation of the Retinoic acid Orphan Receptor γ thymus (RORγt) transcription factor in mouse, or RORC in human, a critical transcription factor (TF) implicated in Th17 cell development [15]. As CD161 surface molecule is induced by RORC, it serves as a marker of human Th17 cells [16]. Then TGFβ renders naive T cells sensitive to IL-23 by increasing expression of its receptor [17]. In the second and third steps, IL-21 participates to Th17 cell expansion, whereas IL-23 stabilizes the Th17 cell phenotype [18]. TGF-β appears to play a pleiotropic role, as it is involved in the generation of regulatory T cells (Tregs) through Forkhead box P3 (FoxP3) expression, but it activates Th17 cell differentiation, in the presence of IL-6. Since IL-6 inhibits FoxP3, but favors Th17 cell differentiation, this results in a reciprocal regulation between Tregs and Th17 cells [19]. In this balance, IL-2 plays also a regulatory role since this growth factor is required for Tregs expansion and activation, but inhibits Th17 cell promotion. Thus, Th17 cells are promoted when IL-2 is consumed, notably by Tregs. At the molecular level, inhibition of IL-17A production is likely to be related to competitive inhibition of STAT-5 binding to the IL-17-enhancer element by STAT-3 [20]. Accordingly, we have reported that interaction between adipose-derived stem cells and T cells promotes Th17 cell activation and IL-17 production through inhibition of STAT5 binding to the IL-17 enhancer element [21]. IRF4 transcription factor seems also involved in RORγt expression, as assessed by the failure of IRF4-deficient T cells to induce RORγt and subsequent Th17 cell differentiation, following IL-6 and TGF-β co-stimulation [22].

### 2.2. Pathogenic Th17 Cells

Th17 cells that have differentiated from naïve T cells in the presence of IL-6 plus TGF-β present limited pathogenicity, as opposed with Th17 cells that have been generated in the presence of IL-1β, IL-6 plus IL-23 with or without TGF-β [23]. The pathogenicity of Th17 cells has been related to their double expression of RORγt and Tbet, leading to double secretion of IL-17 and IFNγ by Th17 cells. Indeed, IFNγ induces pathogenic Th17 cell polarization and recruitment, through induction of IL-1β/IL-23 cytokine secretion by APC, together with CCL20, a chemokine which receptor, i.e., CCR6, is preponderantly expressed by Th17 cells [24]. Moreover, pathogenic Th17 cells are also known to secrete Granulocyte Macrophage-Colony Stimulating Factor (GM-CSF), and to express cytolytic granzyme B, and/or IL-18R [25,26] with IL-18 being able to stimulate IL-17 secretion by Th17 cells [27]. Expression of IL-1R1 is a marker of pathogenic Th17 cells which persists even when Th17 cells lose their ability to secrete IL-17 upon time. It helps thus to distinguish between Th1 cells and “ex-Th17” cells [28]. Finally, pathogenic Th17 cells have been implicated in a number of chronic inflammatory diseases and cancers, as it will be described below.

However, Th17 cells are not the sole IL-17-secreting cells, as mice depleted from functionally CD4^+^ and CD8^+^ T cells only show a 90% reduction in IL-17 secretion [29]. Moreover, RORγt deficient T cell mice display only a 75% deficiency in gut Th17 cells [15].

### 2.3. γδT17 Cells

γδT17 cells are actors of innate immunity, they express TCR with γ and δ chains and produce IL-17. Commitment of γδT17 cell occurs in the thymic compartment, from double negative CD4^−^ CD8^−^ cells whereas Th17 cell skewing occurs in lymphoid organs. γδT17 cells share several surface markers with Th17 cells such as CD161, CCR7, CCR4 and CCR6. They express RORγt transcription factor, and AhR, a marker of non-pathogenic Th17 cells [30]. Like Th17 cells, γδT17 cells secrete IL-17A/F, IL-21 and IL-22, cytokines and express IL-23R [31]. However, in contrast to Th17 cells, γδT17 cells do not require IRF4 transcription factor activation to produce IL-17 [32], and are able to directly respond to cytokine or TLR stimuli, such as TLR1, TLR2 but not TLR4 [30]. Moreover, they classically secrete both IL-17 and IFNγ, through upregulation and activation of both RORC and Tbet TFs. Such an innate response helps to develop immune responses against microbial antigens, such as HIV, or *C*. *Albicans* [33]. Finally, probably due to their ability to secrete both IL-17 and IFNγ, γδT17 cells have also been implicated in autoimmune disorders, such as rheumatoid arthritis (RA) or experimental autoimmune encephalomyelitis (EAE) [34,35].

### 2.4. Invariant Natural Killer T (iNKT) Cells

iNKT cells are enriched in tissue barriers including lung and skin, but are also found in adipose tissues and liver. They belong to the innate immune system and serve to mount an efficient defense against microbial pathogens. iNKT cells express an invariant TCR which recognizes self or foreign glycolipids, such as α-galactoceramide (α-GalCer), loaded on the CD1d molecule (MHC-I like molecule). IL-17-producing iNKT cells (iNKT17) express RORγt and AhR, but do not secrete IL-22. Their characteristic phenotype is CD4^−^ NK1.1^−^ CD161^+^, IL-23R^+^. Like pathogenic Th17 cells, their activation depends on the presence of IL-1β, IL-6, IL-23 and TGF-β which activates the PI3K/AKT/mTOR signaling pathway [36,37]. IL-17^+^ iNKT cells have been shown to drive neutrophilic airway inflammation upon adoptive transfer [38] and have been implicated in diseases, such as T2D [39].

### 2.5. Innate Lymphoid Cells (ILC)

ILC are found in various tissues including intestine, adipose tissues and mesenteric lymph nodes. Moreover, mucosal surfaces are enriched in ILC. They regulate tissue development, integrity and homeostasis [40] and are described as the former innate immune cells able to react against pathogens because of their location in the gut. Of interest is the identification of different subsets, which secretion patterns correspond to those of T helper cells. Thus, ILC1/Th1, ILC2/Th2, ILC3/Th17 cells present highly similar cytokine patterns.

ILC3 are niched in the gut lymphoid follicules, where they can cooperate with various immune cells to induce an efficient and rapid immune response against pathogens. They also express RORγt and CD161. ILC3 predominantly secrete IL-22, which plays an essential role in epithelial and stem cell regeneration, and in homeostasis between host and microbes at mucosal surfaces [41]. IL-17 is secreted by CCR6^+^, but not CCR6^−^ ILC3 cells, in response to bacterial or fungal antigens, through MHC-peptide presentation [42]. Consistent with this observation, ILC3 lacking MHC II fail to regulate host-specific commensal bacteria interactions, but initiate the onset of inflammation, as demonstrated in inflammatory bowel disease (IBD) [43]. Finally, IL-23-secreting dendritic cells (DC) are sufficient to induce IL-17 and IL-22 secretion by ILC3, while IL-7 and IL-1β stimulate proliferation and survival of ILC3 [44]. In pathology, ILC3 can exert pro-or anti-tumor activities depending on the environment and the stage of the disease. ILC3 are increased in peripheral blood of patients with psoriasis [45], and are linked with obesity-associated asthma [46]. A correlation between ILC3 and multiple sclerosis (MS) has recently been reported [47].

## 3. Mechanisms Involved in Th17 Cell Polarization in Obese AT, or Periphery

Obesity has been shown to promote expansion of IL-17-producing T cells in AT or periphery, in human and rodent models, as well. The presence of CD4^+^- and CD8^+^-infiltrating cells in obese adipose tissues is correlated with the occurrence of insulin resistance. In addition, CD4^+^ and CD8^+^ cells are more abundant in visceral AT as compared with subcutaneous AT. Among CD4^+^ cells, both IFNγ^+^/TNFα^+^-secreting cells Th1 cells and Th17 cells have been identified [48]. However, Winer et al., using the diet-induced-obesity (DIO) murine model, have shown a selective promotion of splenic IL-17A^+^ CD4^+^ T cells pools, without modification of Th1 and Th2 pools [49]. Accordingly, a significant increase in circulating IL-17 and IL-23 cytokines has also been observed in obese as compared with lean individuals, in human [50]. The relationship between obesity and Th17 cells has been further demonstrated in a sheep model, where maternal obesity provoked an inflammatory state in fetal intestine, with increased levels of inflammatory cytokines, and in particular IL-17 [51]. Infiltrating IL-17^+^-secreting cells have also been found more abundant, in obese versus lean AT, and in visceral versus subcutaneous AT [52]. Dalmas et al. have shown that single populations of IL-17^+^ IL-22^−^ and IL-17^−^IL-22^+^ and double populations of IL-17^+^ IL-22^+^ CD4^+^ T cells were highly induced in the mucosal tissues of obese subjects, as compared with non-obese. Moreover, they have also demonstrated that T2D further increased the frequency of these cells [53]. Supporting the implication of IL-17 in the metabolic syndrome, the levels of IL-17R expression in liver or muscle were shown to correlate with insulin-resistance [52], and IL-17 blocking resulted in the decrease of hepatic inflammation in the non-alcoholic steatohepatitis syndrome (NASH) [54].

### 3.1. Obesity and Adipose Tissue Inflammation: Role of Macrophages

Besides adipocytes, lean or obese AT contain a wide range of cells such as endothelial cells, fibroblasts, adipose stem-cells (ASC), smooth muscle cells, local mast cells, resident macrophages and leukocytes. Therefore, AT is considered as an endocrine tissue, relative to its aptitude to secrete (i) adipokines, such as leptin or, adiponectin, (ii) cytokines, and/or (iii) chemokines. Adipocyte homeostasis will depend on the balance between adiponectin plus anti-inflammatory cytokines, versus leptin plus pro-inflammatory cytokines. Thus, in the non-obese state (BMI ≤ 25 kg/m²), healthy AT expansion relies to appropriate adipogenesis (hyperplasia), adequate angiogenesis and effective remodeling of the extracellular matrix (ECM). In contrast, sustained increase in energy intake induces pathological AT expansion with massive enlargement of existing adipocytes (hypertrophy) and restricted angiogenesis, resulting in a poorly oxygenated AT [55]. AT hypoxia leads then to adipocyte death and initiates macrophage recruitment [56]. The metabolic health of adipocytes has been correlated with macrophage numbers and their activation state [57]. Namely, in lean AT, depots contain fewer than 10% of resident macrophages, whereas in severe obese individuals, macrophage content can increase up to 50% [58]. Furthermore, in obese AT, 90% of macrophages are localized close to dead adipocytes [59], allowing their aggregation to necrotic adipocytes and formation of crown-like structure (CLS), a histological hallmark of inflammation and insulin resistance, which subsequently leads to AT fibrosis [60]. Moreover, at the qualitative level, macrophages are quite different in lean versus obese AT. Indeed, resident anti-inflammatory, or “alternatively activated” M2 adipose tissue macrophages (ATM) secrete IL-10, IL-4, IL-13, TGF-β and express IL-1RA (IL-1 receptor antagonist), Macrophage Galactose type C-Lectin 1 (MGL1), arginase 1 (Arg1), Ym1 and CD163 [56,60,61]. Obesity induces a switch from anti-inflammatory M2 ATM towards pro-inflammatory M1 ATM which secrete pro-inflammatory cytokines, such as TNF-α, IL-6, IL1-β, IL-12, IL-23, and leukocyte-attracting chemokines, such as IL-8, Rantes, MIP-1α and MIP-1β. Moreover, M1 ATM express CCR2 and CD62L and contribute to angiogenesis and remodeling, through metalloproteinase (MMP) up-regulation, notably MMP9 [62,63]. At the transcriptional level, M1 and M2 ATM are characterized by the expression of IRF5 or IRF4, respectively [64]. Thus, IRF5 deficiency is able to prevent M2 towards M1 switch in DIO-mice, and to results in limited expansion of visceral AT together with enhanced insulin sensitivity [65].

Pro-inflammatory M1 macrophages play thus a crucial role in attracting other immune cells inside AT, such as neutrophils, Th1 and/or Th17 cell subsets.

### 3.2. Macrophages and Th17 Cell Polarization in Obese AT

**Figure 1:**
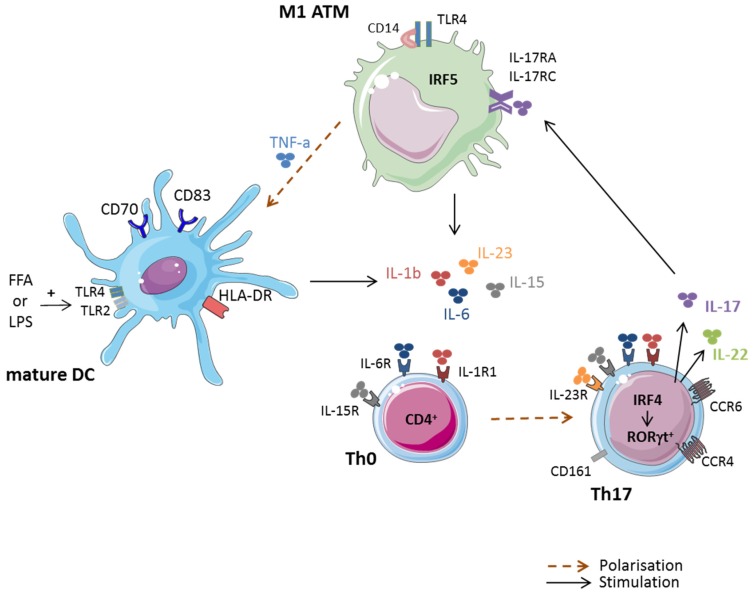
Pro-inflammatory M1 adipose tissue macrophages (ATM) promote IL-17-secreting CD4^+^ Th17 cells in obese adipose tissue (AT). Obese M1 ATM secrete high levels of pro-inflammatory cytokines, including IL-1β, IL-6, IL-23, and IL-15, which are involved in the promotion and maintenance of Th17 cells. Th17 cells can be either induced from naive IL-23R^−^ CD4^+^ T cells in response to IL-1β, IL-6 and IL-15 or resting IL-23R^+^ Th17 cells in response to pro-inflammatory cytokines plus IL-23. Interestingly, CD14^+^ M1 can induce maturation of infiltrating dendritic cells (DC) when stimulated by lipopolysaccharide (LPS) or free-fatty acids (FFA). Mature DC, can lead to Th17 cell skewing, through secretion of IL-1β, IL-6 and IL-23, in obese AT.

The first evidence of the involvement of macrophages in the polarization of T cells towards IL-17 secreting cells was supported by studies demonstrating a correlation between the presence of pro-inflammatory M1 ATM and CD4^+^ Th17 cells in obese AT, as shown in Figure 1. Thus IL-1β, IL-6 and IL-23, which are produced by M1 ATM, have been correlated with skewing and expansion of Th17 cells in obese and T2D patients [66,67]. Using IL-1β blocking experiments in a co-culture model where CD3^+^ and CD14^+^ cells were derived from obese AT, Dalmas et al. have confirmed the role of macrophage-secreted IL-1β in IL-17 production [53]. Indeed, overexpression of IRF5 in M1 macrophages induced activation of genes encoding inflammatory cytokines involved in Th17 cell differentiation or expansion, such as IL-6, and IL-23. TNFα, a cytokine known to be overexpressed in obese AT [7], has been demonstrated to indirectly promote naïve T cells to differentiate into Th1 and Th17 cells as well, through induction of monocyte differentiation into mature dendritic cells (DC) [68] (Figure 1).

In addition to M1 macrophages, a new macrophage subset has recently been identified as able to selectively activate Th17 cells and down-regulate Th1 and Th2 subsets, called mycobacterial infection-induced suppressor macrophages (MIS-MQ). This MIS-MQ subset can be distinguished from M1 and M2 macrophages by its increased levels of IL-1β and TNF-α gene expression [69]. Finally, the clear demonstration of the implication of CD14^+^ monocytes in the maintenance of the Th17 cell cytokine signature in peripheral blood T cells from T2D patients, was demonstrated with the use of CD14^+^ depleted cell populations in CD3^+^/CD14^+^ co-cultures. Indeed, the rate of IL-17 secretion decreased down to the levels of non-diabetic patients [70].

### 3.3. Contribution of ASC and Adipocytes in Th17 Cell Polarization

Besides M1 macrophages, adipose-derived stem cells (ASC) can also polarize infiltrating T cells towards the pathogenic Th17 cells, IL-17/IFNγ double secreting subset, in obese AT. This has been demonstrated by our team, using a co-culture model, with ASC and peripheral blood mononuclear cells (PBMC). In this model, CD14^+^ cells were not found absolutely required, albeit able to amplify ASC-mediated IL-17 production, through IL-1β secretion [21]. Interestingly enough, adipocytes that were differentiated from obese ASC, displayed a similar ability to promote pathogenic Th17 cells [71]. Moreover, the inflammatory cytokine content of these co-cultures was shown to induce insulin resistance of obese ASC-derived adipocytes and to impair ASC adipogenesis, probably related to the presence of IL-17 in the milieu. Accordingly, IL-17 has been shown to downregulate expression of pro-adipogenic TFs, such as PPARγ, C/EBPα and KLF15 and to upregulate expression of anti-adipogenic TFs, such as KLF2 and KLF3, in adipocytes [72]. The anti-adipogenic effect of IL-17A can also be caused by COX-2 activation and subsequent increased levels of prostaglandin E2, as treatment of human mesenchymal stem cells with aspirin, a COX-2 inhibitor, reverted the anti-adipogenic IL-17 effect [73].

### 3.4. Immunometabolism and Th17 Cell Differentiation

Recent studies have pointed out the role of energy in T helper (Th) cell differentiation, expansion and proliferation [74,75]. Thus, metabolic nutrients are likely to regulate the interplay between Th cells and regulatory T cells, relative to their distinct energy requirements. One such example has been clearly demonstrated with Th17 cells and Foxp3^+^ Tregs. Indeed, as detailed above, Th17 cells and Tregs are tightly and reciprocally regulated by the coordinated action of IL-1β, IL-23, plus IL-6, in favor of pathogenic Th17 cells or IL-2 plus TGF-β, in favor of Tregs [19]. However, cytokines appear not to be the sole players in this balance. Glycolysis, which allows the rapid conversion of glucose into pyruvate gives rapid access to 2 ATP molecules, whereas oxidation of pyruvate and subsequent mitochondrial phosphorylation (OXPHOS) are much more potent in fueling the cells, since 36 ATP molecules are generated [76]. Even though less ATP is produced, Th17 cell differentiation and proliferation is preferentially induced by glycolysis, and therefore preferentially uses glucose, amino-acid, or nucleotide substrates, whereas Tregs differentiation is rather induced by the OXPHOS of fatty acid substrates (FAO) [76,77]. As mentioned in a preceding study, hypoxia appears to precede and provoke the onset of inflammation in obese adipose tissues [56]. Such hypoxia increases levels of HIF-1α, an activator of the glycolytic metabolism, which facilitates Th17 cell generation, under the control of mTOR [78,79,80], but inhibits Tregs generation, through up-regulation of the Pyruvate Deshydrogenase Kinase (PDHK1), an enzyme impairing OXPHOS [81]. Accordingly, rapamycin, an mTOR inhibitor has been demonstrated to favor Tregs generation, while inhibiting Th17 cell differentiation [82]. De novo fatty acid synthesis (FAS) is also interfering in the balance between Th17 cells and Tregs, since inhibition of acetyl carboxylase (ACC1), a crucial enzyme for de novo fatty acid synthesis, was demonstrated to favor Tregs and impair Th17 cells, indicating thus that FAS is required in the process leading to Th17 cell promotion [83]. Interestingly, a strong correlation between ACC1 expression and Th17 cell differentiation was found in obese mice [84]. Oxysterols such as 27-dihydrocholesterols, oxidized lipoproteins (oxLDLs) or lisophosphatidic acids (LPA) have also been implicated in the promotion of Th17 cells, contributing thus to the understanding of the impact of obesity and its excessive nutrients in Th17 cell promotion [85,86,87,88].

## 4. Obesity and Th17 Cell-Driven Inflammatory Diseases

### 4.1. Rheumatoid Arthritis

Obesity is likely to be a risk factor of RA, because obese patients display more active and severe RA disease than lean patients. This has been correlated with increased levels of synovial Th17 cells [89]. Accordingly, in the collagen-induced arthritis (CIA) murine model, aggravation of RA in obese mice, as compared with lean mice, has been shown to correlate with higher titers of anti-type-II collagen immunoglobulins, and increased levels of IL-17 mRNA [90]. Moreover, IL-8, a chemokine, involved in the recruitment of neutrophils and up-regulated by IL-17, has been found to be increased in the synovial membranes of obese individuals [91]. Finally, T2D has also been associated with increased risk to develop RA [92].

### 4.2. Multiple Sclerosis

Obesity is reported as a risk factor of MS, since a correlation between BMI, genetic factors, and MS has been reported [93]. This is likely due to a crosstalk between adipose, immune and neural tissues, through adipokine secretion [94]. Indeed, leptin deficient obese mice (ob/ob mice) have been found to be resistant to EAE induction, and to present a Th17 cell deficiency [95]. In contrast, adiponectin deficient mice have been shown to develop severe EAE, with high levels of IL-17, IFN-γ and IL-6 [96].

Chemerin is an adipokine likely to play a role in EAE, through increased chemerin-receptor expression in CNS-infiltrating leukocytes [97]. Interestingly enough chemerin plasmatic levels are up-regulated in obese MS patients [98]. Supporting the role of obesity in MS pathophysiology, five week calorie restriction has been shown to significantly reduce inflammation and demyelination, correlated with increased serum adiponectin and reduced IL-6 and leptin in EAE mice [99]. Interestingly, a drastic reduction in the number of spinal cord Th17 cells and a decrease in IL-17 seric levels have been found in calorie restricted EAE suffering mice. In this study, the authors have also demonstrated a significant reduction in Th1 cytokines and numbers [100]. Finally, besides pathogenic Th17 cells, obese ASC have also been shown to exacerbate clinical symptoms of EAE mice [101], supporting our data about the pro-inflammatory role of ASC in obesity [21].

### 4.3. Psoriasis

Obesity is an additional risk factor of psoriasis [102] possibly due to shared characteristics, such as higher serum levels of leptin and/or VEGF-α and lower levels of adiponectin. At the metabolic level, obesity is associated with elevated serum levels of FFA, IL-17, IL-22 and IL-23 which have been correlated with psoriasis severity [103]. Accordingly, treatment of human keratinocytes with palmitic acid, a fatty acid mainly involved in obesity development, induced the expression of Th17 cell-related cytokines together with Reg3γ, which resulted in epidermal hyperplasia, like in psoriasis [104]. Finally, lipocalin-2 (LCN2), an adipokine correlated with obesity and IR, has been shown to exacerbate psoriatic skin lesions in mice, through increase of IL-17A/F, IL-23p19, IL-12p40, CCL20 and TNF-α, levels, but not IL-12p35 [105]. Altogether, these reports highlight the combined action of FFA and IL-17 in psoriasis aggravation.

### 4.4. Cancer

Various factors that are present in obesity and T2D are able to increase the risk of developing cancer, such as chronic inflammation, metabolic dysfunction and altered gut microbiota. Inversely, energy restriction is associated with reduced tumor development, and refeeding abrogates the protective effects of fasting against cancer development, in rodent [106]. Among obesity-related adipokines, leptin is likely to play a major role in cancer growth. Indeed, the leptin receptor (ObR) is expressed in various human cancers including colorectal, breast, ovarian or prostate cancers [107], and contributes to carcinogenesis, proliferation, and metastasis [108]. Inversely, adiponectin elicits opposite effects, as administration of adiponectin has been shown to decrease proliferation, vascularity, growth, and invasion, and to increase apoptosis of colorectal or prostate cancer cells [107,109]. Thus, obese preadipocyte-, or mature adipocyte-surrounding tumors (also named cancer-associated-adipocytes, CAA) are likely to negatively impact the course of tumor, through increased secretion of leptin or pro-inflammatory cytokines, as demonstrated in human breast cancers [110,111]. ASC have also been implicated in cancer invasiveness, due to their overexpression chemokine receptors (such as CCR3, CCR7, CXCR4). This allows them to migrate towards tumor sites [112] and exert pro-tumoral properties, such as promotion of neo-angiogenesis and expansion of tumor mass [113].

Besides ASC and adipocytes, Th17 cells and IL-17 secreting cells have been found to infiltrate several types of cancers, in human or rodent models, as well [114,115,116,117,118,119]. But their role in cancer prognosis is controversial and seems to be tissue specific or dependent on host immune responses [117,120]. Indeed, several studies have linked IL-17 producing cells with protective effects against tumor development and progression [120,121,122,123,124,125], whereas other studies have correlated the presence of Th17 cells or IL-17-secreting cells in tumors or serum with a poor prognosis [126,127,128,129,130,131,132,133,134].

At the mechanistical level, the anti-tumor activity of Th17 cells is likely to be related to the recruitment of effector cells, such as Th1, CD8^+^ cells, NK cells, or dendritic cells within the tumor micro-environment, through increased secretion of CXCL9, CXCL10, and CCL20 [123,135,136], and/or the negative presence of Tregs [114]. Moreover, pathogenic Th17 cells have been shown to be as more potent effectors than Th1 cells in the eradication of cutaneous melanoma, due to their ability to secrete IFNγ [124]. Accordingly, recent reports have demonstrated that the efficiency of cyclophosphamide in eradicating tumors may be related to the alteration of the microbiota composition in favor of bacterias able to stimulate generation of pathogenic Th17 cells [122,137]. In contrast, the pro-tumoral role of Th17 cells may be related to their ability to induce tumor vascularization, especially in immune-deficient mice [117]. Indeed, IL-17 induces IL-6 production by tumor cells and/or tumor-associated stromal cells, which results in the activation of STAT3, a well-known oncogenic TF, that up-regulates pro-angiogenic factors [138]. Thus, host immune competency is likely to account for Th17 cell role in tumor development.

Table 1 highlights recent studies in which IL-17-secreting cells have been found to display anti- or pro-tumoral properties.

## 5. Conclusions

Obesity is well known to predispose individuals to the metabolic syndrome. The mechanisms involved in obesity-related pathological processes implicate various immune cells, among which pathogenic Th17 cells are likely to play a crucial role, not only through their pro-inflammatory properties, and their ability to propagate inflammation, but also, through their immunometabolism, which can be easily modified with a hypercaloric diet. As it is mentioned before hypercaloric diet induce an enrichment of pathogenic Th17 cells, in several metabolic organs, however it provoke a pronounced decrease of intestinal IL-17/IL-22 secreting RORγt^+^ Th17 cells [139]. In addition, the involvement of Th17 cells in the pathogenesis of autoimmune diseases and cancer, give clues as the reason why obesity is a risk factor in these diseases.

Of particular interest is the role of mesenchymal stem cells (MSCs) in the regulation of the Tregs/Th17 cell balance. Indeed, as demonstrated by our team, or by Patel et al, in breast cancer, MSCs are able to switch from immune-modulatory towards pro-inflammatory functions, depending on their interaction with T cells in lean versus obese AT [21], or with cancer stem cells versus breast cancer progenitors [140]. Thus, identifying the molecules involved in the interaction of MSCs with T cells, or cancer stem/progenitor cells, should help to control the immune-modulatory versus pro-inflammatory cell function of MSC, and therefore the Tregs/Th17 cell balance.

**Figure 2:**
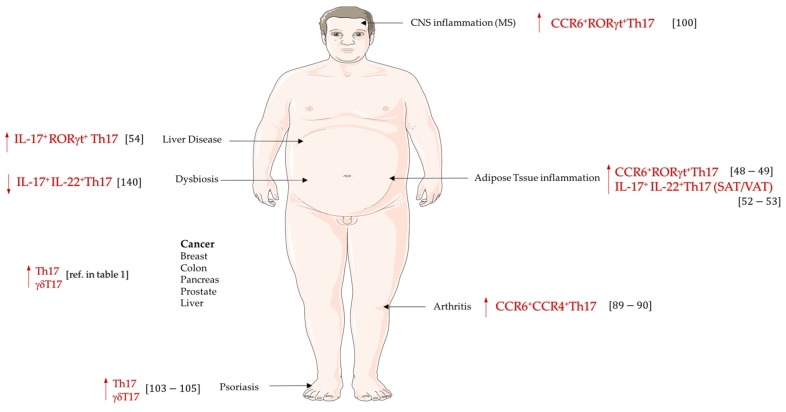
IL-17-secreting cells in obesity related pathologies. A variety of IL-17-secreting cells is implicated in obesity and/or related-inflammatory diseases. Whereas obesity-induced dysbiosis is associated with a decrease in gut Th17 cells, Th17 cell frequency is enhanced in peripheral blood, and metabolic organs, such as liver, pancreas, or adipose tissues, in obesity and/or related-inflammatory diseases.

Figure 2 summarizes the implications of IL-17 secreting cells in various autoimmune or chronic inflammatory diseases linked to obesity.

## Figures and Tables

**Table 1:** IL-17-secreting cells: pro- or anti-tumoral activity? This table highlights different studies in which IL-17-secreting cells were associated with either anti- or pro-tumoral activities.

IL-17 Effects	IL-17 Secreting Cells	Cancer Type	Experimental Model	Key Findings	Ref.
**ANTI-TUMORAL EFFECTS OF IL-17**	**Th17** **Tc17**	MC38 colorectal tumor cells	in vitro assays; cells were SC injected in C57BL/6 mice followed by adoptive transfer of polarized-Tc17 treated or not with LYC-54143	Activation of RORγt with an agonist (LYC-54143) enhanced Th17 (IL-17+-CD4+) and Tc17 (IL-17+-CD8+) effector activity and reduced Treg immunosuppressive afunction, allowing potent anti-tumor response in vitro. Adoptive transfer of Tc17 cells treated with RORγt agonists inhibited tumor growth.	[121]
	**pTh17** **Th1-Th17** **γδT17**	MCA205 fibrosarcoma, MC38-OVAdim colorectal cells	SC injection of tumoral cells into ATBs-treated C57BL/6 mice followed with E. hirae or B. intestinihominis bacteria inoculation	Hirae markedly increased pTh17 cells (a population of double positive IFN-γ+IL-17+ T cells), cytotoxic IFN-γ+CD8+ T cells, but decreased regulatory Foxp3+CD25+CD4+ TILs. B. intestinihominis-inoculated mice increased the numbers of IL-17+γδT cells and IFN-γ+ γδT cells infiltrating tumor. These 2 bacteria species enhanced anticancer immune responses.	[122]
	**pTh17**	MCA205 fibrosarcoma, B16 melanoma	SC injection of tumoral cells into cyclophosphamide-treated SPF mice	The anti-tumor efficacy of cyclophosphamide depended on the translocation of Gram+ bacteria in secondary lymphoid oragans, which stimulated pathogenic Th17 (gut pTh17) generation and memory Th1 immune responses. These results suggest that the gut microbiota help shape the anti-cancer immune response.	[137]
	**Th17**	B16 lung melanoma	IL-17 deficient mice bearing B16/F10 melanoma	Adoptive transfer of tumor-specific CCL20+ -secreting Th17 cells inhibited tumor growth through improvement of tumor antigen presentation to dendritic cells (CCR6+CD11c+CD11b+ and CCR6+CD11c+CD8α+ DC), and amplification of tumor-specific CD8+ T cell killing activity. Th17 (75% fewer tumor colonies) had a greater potency to control tumor growth than Th1 (40% fewer tumor colonies) when transferred into mice harboring melanoma.	[123]
	**Th17**	B16 melanoma	SC injection of tumoral cells into TRP-1 TCR transgenic mice	Among the Th0, Th1, and Th17 subtypes, it was found that Th17-polarized cells better mediated destruction of advanced B16 melanoma. However, their therapeutic effect was critically dependent on interferon-gamma (IFNγ) production.	[124]
	**γδT17**	B16/F10 melanoma and lung Lewis carcinoma	IV injection of tumoral cells in ATBs-treated C57BL/6 mice after adoptive transfer of γδT cells	Antibiotics increased susceptibility to develop engrafted B16/F10 melanoma and Lewis lung carcinoma in mice. Adding normal γδT cells or supplementing IL-17 restored the impaired immune surveillance phenotype, which demonstrated the importance of commensal bacteria in supporting the host immune response against cancer.	[125]
	**Th17**	Ovarian cancer	Human	Levels of tumor-infiltrating Th17 cells are negatively correlated with tumor-infiltrating regulatory T cells. Th17 cells contributed to protective human tumor immunity by inducing Th1-type chemokines, CXCL9 and CXCL10, and recruiting effector cells to the tumor microenvironment.	[135]
**PRO-TUMORAL EFFECTS OF** **IL-17**	**γδT17**	Hepa1–6 hepatocellular carcinoma cell line	SC injection of tumoral cells in C57BL/6 or IL-17^−^/^−^C57BL/6	The tumor-promoting effect of IL-17A, mainly produced by γδT17 cells, was mediated through suppression of antitumor responses, especially CD8^+^ T cell responses. IL-17A induced CXCL5 production by tumor cells to enhance the infiltration of myeloid-derived suppressor cells (MDSC) to tumor sites in a CXCL5/CXCR2-dependent manner	[130]
	**Th17** **Tc17**	Hepatocellular carcinoma (HCC)	Human	A cohort of 105 patients with liver cirrhosis and an established HCC revealed higher serum levels of IL-17 and a massive hepatic infiltration of CD4^+^ and CD8^+^ IL-17 secreting cells	[131]
	**Th17**	HCC	High Fat Diet fed C57BL/6 mice	Hepatic unconventional prefoldin RPB5 interactor (URI) induced enriched diet to cause liver DNA damages, recruitment of Th17 cells in liver, development of a non-alcoholic steatohepatitis (NASH) and of HCC. pharmacological suppression of Th17 cell differentiation, IL-17A blocking antibodies, and genetic ablation of the IL-17A receptor in myeloid cells, prevented diet induced Th17 and subsequent HCC.	[126]
	**Th17**	CRC (Colorectal Cancer)	Human	Elevated numbers of CD4^+^ Th17 in 54 CRC patients were observed inside tumors compared with non-tumor regions, together with a higher microvessel density. Colorectal cell lines stimulated IL-17 produced VEGFα in a dose dependent manner	[127]
	**IL-17 and TNF-α secreting Th17**	CRC (HCT116) and prostate (LNCaP) cancer cell lines	Human	IL-17 and TNF-α individually rather than cooperatively, up-regulated PD-L1 expression in human prostate and colon cancer cell lines. PD-L1 expression acts on PD-1 ligands (PD-L1 and PD-L2) to suppress activation of cytotoxic T lymphocytes.	[132]
	**IL-17 and TNF-α secreting Th17**	CRC	human	An increased frequency of Th17 cells was observed inside 22 CRC tumor tissue, as compared to adjacent uninvolved tissue. These Th17 cells mostly coproduced TNF-α, but not IFN-γ. There was a negative correlation between expression of PD-1 and IFN-γ, but not IL-17, in CRC, and an enrichment in Tregs. Thus PD-1 expressing T cells and Treg cells within the tumor may have a suppressive effect on T cells secreting IFN-γ, IL-2, or TNF-α, but not Th17 cells	[128]
	**IL-17 and TNF-α cytokines**	CRC cell lines (Caco-2, HCT116)	Human	IL-17 and TNF-α were shown to enhance glycolysis in several colorectal cancer cell lines, through increased expression of HIF-1α and c-myc. TNFα and IL-17 also synergistically stimulated production by HT-29 cells of a growth factor that simulated proliferation/survival of NIL8 fibroblastic cells, promoting thus colorectal tumorigenesis	[133]
	**γδT17**	CRC	Human	γδT17 were found predominant in CRC tissues of 154 patients. γδT17 cells promoted migration, survival and proliferation of MDSC via production of IL-8, GM-CSF and TNF-α in vitro	[134]
	**RORγt^+^ Th17**	Prostate cancer	Pten-null mice	The treatment of Pten-null mice with SR1001, Th17 cell inhibitor, or an anti-IL17 monoclonal antibody during 6 weeks was sufficient to reduce prostate tumor progression, angiogenesis and tumor cell infiltration.	[129]
	**Th17**	B16 melanoma MB49 bladder carcinoma	SC injection of tumoral cells into WT or IL17−/−mice	IL-17 promotes tumor growth through an IL-6/Stat3 pathway	[138]
